# Dynamics of cortical oxygenation during immediate adaptation to extrauterine life

**DOI:** 10.1038/s41598-021-01674-9

**Published:** 2021-11-11

**Authors:** Léa Leroy, Mahdi Mahmoudzadeh, Jean Gondry, Arthur Foulon, Fabrice Wallois

**Affiliations:** 1 INSERM U1105, GRAMFC, Université de Picardie Jules Verne, CURS, Amiens, France; 2 INSERM U1105, GRAMFC, Department of Exploration Fonctionnelles du Système Nerveux Pédiatrique, Picardie University Hospital, Amiens, France; 3 Department of Gynecology and Obstetrics, Picardie University Hospital, Amiens, France

**Keywords:** Neonatology, Neurophysiology

## Abstract

The neonatal transition involves physiological modifications as a consequence of the complexity of the perinatal period. Various strategies can be used to attain the same level of postnatal cerebral oxygenation, depending on the status of the infant at birth. We evaluated such strategies by recording 20 full-term newborns by near-infrared spectroscopy during the first 10 min of life. The acid–base status at birth revealed two clustered profiles of cerebral oxygenation dynamics. Lower pH and base excess and higher lactate levels were associated with more rapid attainment of the 95% maximal tissue oxygenation index value. These results suggest that metabolic mechanisms drive initial cerebral oxygenation dynamics during this critical period. These results confirm the capacity of newborns to develop multiple strategies to protect the brain.

## Introduction

Birth is a transitional at-risk situation in which the fetus passes from its intrauterine environment to become a neonate in its extrauterine environment. This transition suggests the existence of well-coordinated complex adaptive physiological mechanisms modulated by the history of the pregnancy and possible vascular complications, as well as the labor and its management and the delivery procedures. Fifteen percent of newborns require medical support at birth^1^. Six to 28% of neonatal encephalopathies are the consequence of cerebral hypoxia/anoxia during childbirth^2^. Despite an increase in the number of surgical deliveries, the rate of neonatal encephalopathy associated with cerebral anoxia has remained stable for years^3^. It is thus still necessary to develop innovative approaches to improve the identification of specific risk factors of neonatal hypoxia/anoxia to allow clinicians to intervene upstream of brain tissue alterations.

Oxygenation of the fetus relies on the passage of maternal blood through the placenta to the fetal circulation. This results in low blood oxygen saturation of the fetal tissues. The fetus is thus considered to be in a situation of chronic physiological hypoxia^4^. Nonetheless, the oxygenation status is well adapted for harmonious development inside the womb due to the high oxygen affinity of fetal hemoglobin. After delivery, cord clamping triggers the interruption of communication between maternal and fetal circulation, leading to the closure of physiological shunts, drastically modifying fetal circulation and halting the supply of maternal oxygen to the fetus^5^. At the same time, efficient pulmonary function begins after the first cry, providing external oxygen as the sole source of oxygenation^6^. Overall, these general mechanisms result in increased oxygenation in the systemic circulation^7^, leading to an increase in vascular resistance^8^.

Based on the recommendations of the European Resuscitation Council and the International Liaison Committee of 2021^9^, standardized management of resuscitation in newborns has been developed, taking into account clinical (color, respiration) and paraclinical (cord gas measurements, pre-ductal pulse oximetry, and electrocardiography) factors.

Medical decisions concerning the neonate are clinically based on the Apgar score, developed in 1952. It consists of the evaluation of the color of the skin, the heart and respiration rate, and the tonus and reflexes, providing information about the efficiency of spontaneous breathing, which is essential for the immediate transition at 1 min. The dynamics of the Apgar score between 1 and 10 min^10^ and, notably, the value at 5 min^11^, not only reflect the newborn's condition at birth but also the adaptation of the newborn infant to extrauterine life immediately afterwards. An Apgar score < 7 at 5 min can be considered as a predictive index of neonatal dysfunction. Nevertheless it is generally accepted that the Apgar score lacks objectivity^12^.

A blood sample from the umbilical artery allows objective paraclinical cord gas measurements^13^ (pH, pCO_2_, base excess [BE], and lactate level) to identify respiratory and/or metabolic acidosis, indicative of per partum asphyxia, likely to alter the adaptation to extrauterine life and the later neurological outcome^14^. Acidosis is classified as metabolic, respiratory, or combined based on the measurement of lactate (for the metabolic component) and pCO_2_ (for the respiratory component). An evaluation of the acid–base status based on cord gas is routinely performed to define the impact of the antenatal period and the acid–base status just before the transition.

Pre-ductal arterial saturation^15^ can be monitored as a measure of the dynamics of arterial oxygenation, in particular, cerebral arterial oxygenation. However, cerebral tissue oxygenation may already be optimal before arterial oxygen saturation is complete^16^.

It is therefore necessary to develop new approaches to monitor the strategies of the neuronal and vascular compartment during this at-risk transitional period. Ideally, such an approach should be minimally invasive. Near-infrared spectroscopy (NIRS) offers the opportunity to monitor the cerebral tissue oxygenation index (TOI), which reflects the relative oxygen content within the superficial layers of the cortex. Differences between the dynamics of systemic and cerebral oxygenation after delivery^17^ suggest that NIRS may be useful for specifically evaluating cerebral oxygenation during the neonatal transition^18^.

Two periods have been defined: period 1, during which the hemodynamics rapidly change, and period 2, during which they reach a plateau level^19^. During period 1 (approximately 7 min), cerebral tissue oxygenation and systemic oxygen saturation increase but with different dynamics^10^. Such a difference in the dynamics supports the decrease in fractional cerebral tissue oxygen extraction observed in the first minutes after delivery^20^, likely corresponding to a change in cerebral oxygen demand. Simultaneously, cerebral tissue oxyhemoglobin ([ΔHbO]) increases, whereas tissue deoxyhemoglobin ([ΔHbR]) decreases^17^, along with cerebral blood flow and the cerebral metabolic rate of oxygen^21^. During the circulatory transition, systemic vascular resistance increases, leading to a decrease in cerebral blood flow^22^, along with a decrease in cerebral blood volume ([ΔHbT])^23^. During period 2 (after 7 min), all parameters reach a plateau.

Although the general dynamics have been well described at the group level, published studies lack the individual analyses that would allow delineation of the strategies of the neurovascular system in its ability to adapt to extrauterine life based on available clinical and paraclinical parameters, such as the duration of labor and expulsive efforts, and markers of antenatal asphyxia (acid–base status)^24,25^. Studying cerebral oxygenation immediately after birth is necessary to better understand the neonatal transition to extrauterine life and define eventual predictive biomarkers. The hemodynamic profiles recorded just after birth should be considered to result from multiple factors, including what has occurred in utero and during delivery, and the initial adaptation to extrauterine life.

We evaluated the individual hemodynamic strategies used to adapt to extrauterine life by analyzing the evolution of individual changes in cortical oxygenation parameters through NIRS measurements. A clustering procedure was applied to identify the various strategies of the neurovascular system to adapt to extrauterine life based on the initial pH and lactate and BE levels, which reflect the initial metabolic environment of the neonates before the transition to extrauterine life.

We addressed the issues of whether neonates use different strategies for cerebral transition, whether they depend on metabolic mechanisms, and whether such knowledge can be useful for the management of resuscitation by recording full-term infants using cerebral functional NIRS measurements during the immediate transition. Hence, we studied and analyzed the dynamics of hemodynamic parameters to search for correlations with antenatal, prenatal, and postnatal factors.

## Results

### Clinical data

Thirty newborns were initially included in this study (eight were excluded due to parents’ refusal during recording). Two were excluded because they required respiratory support. Among the remaining 20 newborns (Table 1), 14 were vaginally delivered, three instrumentally delivered, and three born by planned Caesarean section (Table 2). The median GA was 39.5 weeks [38.8–40.8]. The median birth weight was 3390 g [3000–3600]. The median pH was 7.28 [7.25–7.31]. Median lactate was 2.7 [2.1–3.52], and median BE − 2.3 [− 4.4– − 0.6]. The median time between delivery and the beginning of recording was 1.75 min [1.25–2.37]. Each newborn adaptation to extrauterine life was clinically evaluated. The Apgar score was normal in all cases (10/10/10 at 1, 5, and 10 min, respectively).

**Table 1:** Clinical features of the tested infants.

Subject ID	Gender	Gestational age (wGA)	Birth weight (g)	Birth weight (percentile)	pH	BE (mmol/L)	Lactates (mmol/L)	pCO_2_ (mmHg)	Risk for infection
sub_01	M	40.57142857	2910	7	7.3	− 0.5	2.1	52.8	Maternal carriage of Streptococcus B
sub_02	M	38.85714286	3710	79	7.35	− 0.6	2.4	45.1	Rupture of membranes > 12 h
sub_03	M	41.14285714	3400	32	7.28	− 0.9	3.7	55.7	–
sub_04	F	41.57142857	3190	25	7.25	− 4.4	3.6	51.2	–
sub_05	F	41.71428571	3945	83	7.31	− 6.9	5.4	37.2	Rupture of membranes > 12 h
sub_06	M	41.85714286	3270	20	7.26	− 3.5	3.5	53.4	–
sub_07	F	38.71428571	2900	21	7.21	− 1.8	3	64.6	–
sub_08	F	40	3810	85	7.38	− 0.6	1.2	41.1	–
sub_09	M	39.28571429	3650	80	7.3	− 0.3	1.7	54.1	–
sub_10	F	39.28571429	2530	5	7.24	− 0.4	2.1	64.2	–
sub_11	F	40.14285714	3600	75	7.34	− 4.4	1.7	38.3	Rupture of membranes > 12 h
sub_12	M	37.14285714	2630	30	7.29	− 2.4	2.1	50.2	Rupture of membranes > 12 h
sub_13	F	38.71428571	3030	30	7.28	− 2.2	2.1	51.3	–
sub_14	F	38.85714286	3200	50	7.25	− 2.9	3.4	56.3	Maternal carriage of Streptococcus B
sub_15	M	38.71428571	3420	50	7.33	− 4.1	3.1	40	Rupture of membranes > 12 h
sub_16	F	39	2530	3	7.29	− 1.9	2.1	51.9	Maternal carriage of Streptococcus B
sub_17	F	40.71428571	3454	50	7.18	− 5	4.9	42.7	Rupture of membranes > 12 h
sub_18	F	41.42857143	3470	52	7.25	− 5.3	3.4	49.4	Maternal carriage of Streptococcus B
sub_19	M	39.57142857	3600	65	7.36	− 0.3	1.2	44.4	Rupture of membranes > 12 h
sub_20	M	39.42857143	3380	45	7.16	− 8.2	6.6	58.3	Rupture of membranes > 12 h

*M* male, *F* female.

**Table 2:** Clinical features of the tested infant’s mothers.

Subject ID	Age (year)	Complications of pregnancy	Parity	Spontaneous/induced labour	Fetal heart rate abnormalities	Delivery	Assisted delivery reasons	Fetal presentation	Amniotic fluid	Labour duration (minutes)	Duration of expulsive efforts (minutes)
sub_01	34	–	5	Spontaneous	Early recurrent decelerations	SVD	–	Cephalic	Clear	4	3
sub_02	33	–	1	Spontaneous	–	SVD	–	Cephalic	Clear	7	3
sub_03	37	–	5	Spontaneous	–	SVD	–	Cephalic	Clear	2	5
sub_04	19	GD	1	Spontaneous	–	VAD	IEE + DEE > 30 min	Cephalic	Clear	9	25
sub_05	23	–	1	Spontaneous	Variable decelerations	SVD	–	Cephalic	Stained	14	35
sub_06	26	–	1	Induced	–	VAD	IEE + FHR abnormalities	Cephalic	Clear	6	11
sub_07	25	–	2	Spontaneous	–	SVD	–	Cephalic	Clear	3	3
sub_08	27	–	3	Spontaneous	–	SVD	–	Cephalic	Clear	2	3
sub_09	33	–	3	–	–	C-section	–	–	Clear	–	–
sub_10	44	SGA	3	–	–	C-section	–	–	Clear	–	–
sub_11	31	–	2	Spontaneous	–	SVD	–	Cephalic	Clear	4	8
sub_12	26	–	1	Induced	–	SVD	–	Cephalic	Clear	7	10
sub_13	30	GD	3	–	–	C-section	–	–	Clear	–	–
sub_14	33	–	2	Spontaneous	–	SVD	–	Cephalic	Clear	4	9
sub_15	34	–	1	Induced	–	SVD	–	Cephalic	Clear	19	10
sub_16	19	–	1	Spontaneous	Variable decelerations	SVD	–	Cephalic	Clear	4	5
sub_17	31	–	1	Spontaneous	Melchior's classification type 2	SVD	–	Cephalic	Stained	5	25
sub_18	15	–	1	Spontaneous	–	VAD	IEE + DEE > 30 min	Cephalic	Stained	10	40
sub_19	30	–	2	Spontaneous	–	SVD	–	Cephalic	Clear	5	1
sub_20	23	–	1	Spontaneous	Variable decelerations	SVD	–	Cephalic	Clear	10	38

*GD* gestational diabete, *SGA* small for gestational age, *SVD* spontaneous vaginal delivery, *VAD* vacuum-assisted delivery, *IEE* inadequate expulsive efforts, *DEE* duration of expulsive efforts.

### Evolution of the hemodynamic parameters for the entire population

After artefact rejection (please see SM for more information), two analyses were applied to the various hemodynamic parameters. The first was an ANOVA, which confirmed the evolution of all hemodynamic parameters during the first 10 min, except for [ΔHbO] (Fig. S2). After applying the Friedman test, TOI (*p* < 0.001), [ΔHbR] (*p* < 0.001), [ΔHbD] (*p* < 0.001), and [ΔHbT] (*p* < 0.05) showed a significant difference over time, in contrast to [ΔHbO] (Table S2).

A period-by-period comparison using the Durbin–Conover test showed a significant difference between several period pairs (Table S3). The TOI showed a significant difference between M1 and M3–M10 (*p* < 0.05), M2 and M3–M10 (*p* < 0.05), M3 and M4–M10 (*p* < 0.05), and M4 and M9–M10 (*p* < 0.05). The [ΔHbR] showed a significant difference between M1 and M4–M10 (*p* < 0.05) and M2 and M4–M10 (*p* < 0.05). The [ΔHbD] showed a significant difference between M1 and M3–M10 (*p* < 0.05), M2 and M3–M10 (*p* < 0.05), M3 and M5–M7 (*p* < 0.05), and M4 and M6 (*p* < 0.05). Finally, the [ΔHbT] showed a significant difference between M1 and M8–M10 (*p* < 0.05), M2 and M9 (*p* < 0.05), M3 and M8–M9 (*p* < 0.05), M4 and M8–M9 (*p* < 0.05), and M5 and M9 (*p* < 0.05).

We further compared the values for all hemodynamic parameters to a reference value to investigate more subtle changes during their evolution.

**Figure 1:**
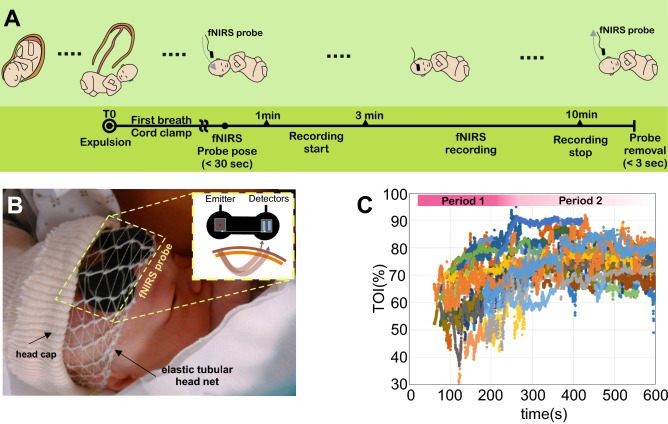
Recording procedures. (**A**) Experimental protocol. (**B**) Set-up for positioning the NIRS probe on the forehead of the newborn. (**C**) Raw TOI data corresponding to all subjects.

Globally, we identified two phases for each hemodynamic parameter. Phase 1 consisted of a rapid increase/decrease in TOI and [ΔHbO], increase in [ΔHbD], and decrease in [ΔHbR] and [ΔHbT]. Phase 2 consisted of a plateau for all parameters (Fig. 1).

**Figure 2:**
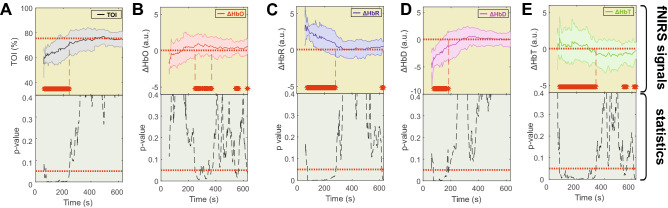
The hemodynamic parameters TOI, [ΔHbO], [ΔHbR], [ΔHbD], and [ΔHbT] are presented during the recording as the mean ± std in panels (**A**) to (**E**), respectively. The red dotted line shows the reference value in each panel. The red bar corresponds to the time window in which the parameters were verified to be significant. The statistical results are presented below each panel. The dotted red line shows the statistical threshold (*p* = 0.05).

The changes in TOI for all newborns were associated with an initial rapid increase (phase 1) followed by a plateau (phase 2) within 300 s. After data averaging and considering the reference period as the mean value between 430 and 630 s, which corresponds to a final TOI of 75.5%, the rapid increase in TOI was significant (*p* < 0.05) from the onset of the recording until 244.2 s (Fig. 2A).

The changes in [ΔHbO] and [ΔHbR] for all newborns were associated with an initial increase in [ΔHbO] and an early decrease in [ΔHbR] (phase 1), followed by a plateau (phase 2) for both conditions. More precisely, [ΔHbO] first showed a non-significant increase until it reached the baseline (t = 248.6 s). From then, [ΔHbO] continued to increase between 248.6 and 369.2 s, at which point the change became significant (*p* < 0.05) (Fig. 2B), whereas the decrease in [ΔHbR] was significant (*p* < 0.05) from the onset of the recording until 282 s (Fig. 2C).

The changes in [ΔHbD] followed the same pattern as the TOI for all newborns, with an initial significant increase in [ΔHbD] from the onset of the recording until 183.2 s (phase 1), followed by a plateau (phase 2) (Fig. 2D). Although the [ΔHbT] curves did not show an initial rapid change, the values of [ΔHbT] significantly (*p* < 0.05) increased from the onset of the recording until 339.2 s (Fig. 2E).

### Clustering of the TOI trajectories

**Figure 3:**
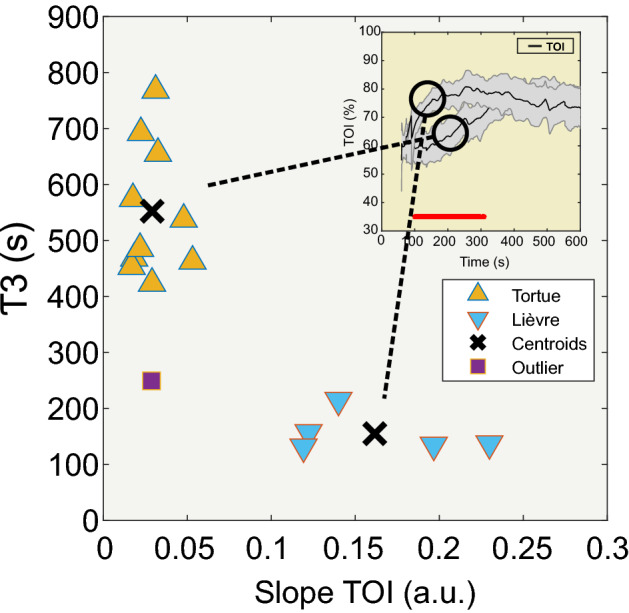
Two-dimensional clustering results are based on the parameters of the slope of the TOI and τ3. We observed two distinct clusters, identified as “Lièvre” (in blue, n = 5) and “Tortue” (in orange, n = 10). The inset presents the average TOI curves (with std) corresponding to each cluster.

**Figure 4:**
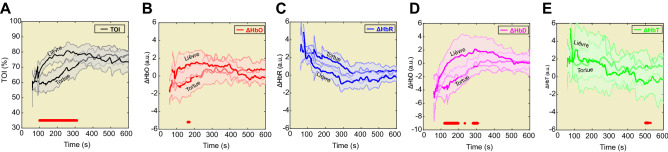
The hemodynamic parameters TOI, [ΔHbO], [ΔHbR], [ΔHbD], and [ΔHbT] are separately presented for each cluster in panels (**A**) to (**E**), respectively. The red bar shows the time window in which the difference between the two clusters was verified to be significant (*p* < 0.05).

The time for the TOI to reach 95% of its maximal value (τ3) showed a bimodal distribution when plotted against the slope of the TOI measured between τ1 and τ2 (Fig. 3). Briefly, when the slope of TOI was small, the time to τ3 was high. Conversely, when the slope was high, the time to τ3 was low. These data support the clustering analysis, which clearly returned two independent clusters. These two clusters were defined as the “Tortue” (Turtle) and the “Lièvre” (Hare) groups (*Fables de la Fontaine “Le lièvre et la tortue”. J De La Fontaine (1668), ed. Claude Barbin. Paris*). Indeed, the fastest increases (slope > 0.1%/s) correspond to the “Lièvre” group (n = 5) and the slowest to the “Tortue” group (n = 10). The difference between the “Tortue” and “Lièvre” groups was significant for the TOI only during phase 1 (Fig. 4). Both groups reached comparable TOI values at the end of the measurement of approximately 75.5%. Note, that the variability was maximal for τ3 for the “Tortue” group, whereas it was maximal for the TOI slope in the “Lièvre” group, suggesting a different strategy to reach the final, similar TOI value.

Although similar profiles were observed for the other hemodynamic parameters ([∆HbO], [∆HbR], [∆HbD], and [∆HbT]) between the two groups, the differences did not reach significance (Fig. 4).

### Identification of predictive factors

**Figure 5:**
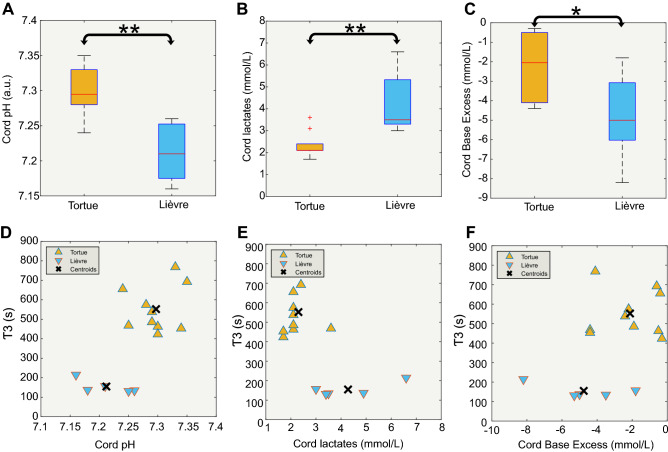
(**A** to **C**) Boxplots presenting the statistical differences between the two clusters, “Lièvre” (n = 5) and “Tortue” (n = 10) in cord gas, cord pH, cord lactates, and cord base excess. (**D** to **F**) Two-dimensional clustering results are presented based on τ3 and cord pH (**D**), τ3 and cord lactates (**E**), and τ3 and cord base excess (**F**).

Among the various parameters used for the identification of predictive values, only cord pH, lactic acid, and BE showed statistically significance differences between the “Lièvre” and “Tortue” groups. (Fig. 5). The pH and BE were significantly higher (*p* < 0.005 and *p* < 0.05, respectively) in the “Tortue” than “Lièvre” group, whereas the level of lactic acid was significantly (*p* < 0.007) lower.

We then examined whether there was a bimodal distribution between the clinical and hemodynamic data. We observed two independent clusters when pH, lactate, and BE were plotted against τ3 (Fig. 5) but not when they were plotted against the slope of the TOI.

The other clinical parameters (maternal age, parity, duration of labor and expulsive efforts concerning the mother, gestational age, and birth weight for the infant) did not show any significant differences between groups.

## Discussion

In this study, assessment of the dynamics of changes in brain oxygenation (i.e., TOI) of healthy full-term neonates immediately postpartum highlights distinct cerebral hemodynamic strategies strongly linked to the initial values of pH, BE, and lactates. The regulation of blood flow in newborns depends on many parameters. Recruitment of capillaries and the extraction of oxygen plays a fundamental role in the regulation of cerebral blood flow, as well as other systemic factors, such as blood pressure, cardiac output, central venous pressure, systemic vascular resistance, partial pressure of oxygen and carbon dioxide in the arteries, postnatal and gestational age, and the presence of patent ductus arteriosus (PDA).

*Regulation of neonatal cerebral hemodynamics:* We focused on regional cerebral hemodynamic parameters, excluding systemic data parameters, which have already been documented during this specific period^19,26^, to limit the number of recordings in this fragile population and in consideration of practical and ethical issues. Three principal mechanisms play an essential role in providing the required amount of oxygen to the brains of newborns in situations of fundamentally decreased blood flow or increased brain activity: first, ***cerebral autoregulation***, which provides the main protection to the brain when the pressure increases or decreases^27^; second, ***cerebral vasomotor reactivity***, which adjusts the hemodynamics to the blood vessel pH/pCO_2_/pO_2_ of the brain tissue, reflecting its oxygen requirement^28,29^; and third, ***neurovascular coupling (NVC)***, which adjusts the perfusion to increased metabolic demand as a result of increased neuronal activity^30,31^. Peripheral oxygenation dynamics follow the same profile^10^ as cerebral hemodynamic changes but with different timing, and cerebral oxygenation may come first^20,32^. Such temporal differences may result from immature protective autoregulation mechanisms and neonatal tolerance to systemic hypoxia^4^.

*Neonatal cerebrovascular strategy to adapt to extrauterine life:* The strategy developed by the neuronal and vascular systems to adapt to extrauterine life does not depend on the mode of delivery^33^. After grand averaging, our hemodynamic results are completely in accordance with those of a previous study^19^, which demonstrated an initial increase in TOI associated with a non-significant increase in [∆HbO], but poorly comparable to the results of a study of Fauchère et al.^17^, and a decrease in [∆HbR] within the first 6 min, followed by a plateau toward the stable TOI value, which is reached in 10 min^17^.

We intended to evaluate whether all patients followed the same strategy to reach normal oxygenation at 10 min after birth or, using a metabolic index, whether we would be able to identify different strategies for the adaptation to extrauterine life related to the past and present clinical history.

Clustering clearly identified two groups of neonates based notably on the dynamics of the initial slope of the TOI, corresponding to the strategy used to adapt to extrauterine life. In addition, these two groups, or strategies, are strongly associated with the initial pH, BE, and lactate levels, which are metabolic indices that result from what has occurred in utero and during delivery.

We, therefore, defined two groups: (1) the “Lièvre” group, which more rapidly reached 95% of its maximum TOI value (τ3), with a steeper TOI slope, higher lactate and lower pH and BE values, and (2) the “Tortue” group, which took longer to reach τ3, with a less steep TOI slope and lower lactate and higher pH and BE values. These metabolic measurements were performed before cord clamping and correspond to the overall metabolic output of all previous events.

The correlation observed in this study between these metabolic indices (pH, BE, lactate) and TOI dynamics in the two groups of neonates suggest different adaptative mechanisms according to the initial degree of hypoxic “stress”. During labor, notably during the expulsive phase, the lack of oxygen due to uterine contractions and abnormal fetal heart rate disturbs the acid–base balance^34^, resulting in acidosis, which reflects the adaptative mechanisms to maintain oxygenation^35,36^. Given the limited oxygen extraction, the cells switch to anaerobic metabolism^37^.

In addition, the first period is essential for metabolic adaptation to the neonatal transition, which is associated with the recruitment of small arteries, which decreases arterial pulmonary blood pressure, in turn facilitating respiratory exchange^38^. This may participate in the different strategies observed between the “Lièvre” and “Tortue” groups to reach the plateau of phase 2.

Fetal cerebral NIRS recordings just before delivery showed a significant correlation between fetal cerebral oxygenation and cord gas at birth^39^. The fetal TOI positively correlated with cord pH at birth and negatively with the base deficit. According to our results, this may suggest that the “Lièvre” group had an initial TOI value inferior to that of the “Tortue” group (Fig. 5). In such a case, the slope of the TOI in the “Lièvre” group to reach the normal plateau TOI value may be steeper.

*Correlation between neonatal cerebral oxygenation and pCO*_*2*_*/pH:* The fetus is in a situation of chronic hypoxia^40^, confirmed by initial TOI values of 46% just before delivery^39^. In this situation, the increase in TOI would correspond to an adaptation to extrauterine life resulting from the closure of physiological shunts, such as the ductus arteriosus and initiation of air-breathing, which generates arterial oxygen enrichment (increase in pO_2_ and decrease in pCO_2_)^8^. This explains the generally reported increase in oxygen saturation, as well as the increase in [∆HbO] and decrease in [∆HbR]^17^, with a slight decrease in [∆HbT] observed here and in other studies^41^, which is less dependent on (correlated with) the initial metabolic conditions. This is compatible with a slight decrease in blood volume^42^ and the vasoconstriction associated with the increase in pO_2_^43^. The same holds true for the increase in SpO_2_, the peak of which may be shifted from that of the increase in cerebral TOI^44^.

The first phase is essential for adapting the metabolism of the newborn to the neonatal transition, which is associated with the recruitment of small arteries, decreasing arterial pulmonary blood pressure and, in turn, facilitating respiratory exchange^38^. This may participate in the different strategies observed between the “Lièvre” and “Tortue” groups to reach the plateau of phase 2.

The correlations observed in the “Lièvre” and “Tortue” groups may also be explained by the Bohr effect. According to the Bohr effect, a decrease in the affinity of hemoglobin for oxygen (O_2_) is observed during an increase in the partial pressure of carbon dioxide (CO_2_) or a decrease in pH, thus facilitating oxygen transfer from blood to tissues. As tissue oxygenation measured by NIRS is mainly dependent on the venous compartments, TOI should decrease in cases of higher extraction. In the fetus, which is in a situation of chronic hypoxia and relative vasodilation, the arteriovenous ratio at the origin of the NIRS signal may be altered in favor of the arterial compartment. This is observed (1) in low pH situations, in which vasodilation is associated with an increase in TOI, especially after surgery for congenital heart disease^45^. Therefore, both low pH and low pO_2_ may interact with the relative preponderance of the arterial or venous compartment at the origin of the NIRS signal. At time t, the TOI in the brain may therefore be higher in a situation of low pH or high lactate. Thus, the pH in the “Lièvre” group was significantly lower and the TOI significantly higher than in the “Tortue” group in the window of 100 to 300 s. Conversely, the “Lièvre” group showed a situation of relative acidosis, likely to induce a decrease in neuronal excitability and favor a decrease in oxygen consumption, resulting in a decrease in oxygen extraction^20^ and, thus, when considering cerebral blood flow, a decrease in the cerebral metabolic rate of oxygen, facilitating a relative increase in cerebral TOI^21^. In accordance with the results of Schwaberger et al.^23^, the increase in the arterial partial pressure of oxygen results in an increase in cerebral vascular resistance and thus a decrease in cerebral blood flow. Consistent with these observations, aEEG recordings indicate decreased cerebral activity during the first 5 min after birth, which then stabilizes. Moreover, low cerebral activity correlates with a low TOI and high oxygen extraction related to normal peripheral oxygen values. Given the differences in the hemodynamics between the two groups, it is possible that the initial vasodilation in the “Lièvre” group is maintained because of a low pH, whereas the reactive vasoconstriction in the “Tortue” group is quicker in response to the increase in PaO_2_ when the pH is higher and pCO_2_ lower. This would result in a delayed decrease in CBV in the “Lièvre” group because of the different metabolic status. The short and small overshoot of TOI and [∆HbD] may therefore represent the consequences of such a delay in vascular reactivity and the different initial TOI dynamics. To a lesser extent, the letter to the editor written by Schwaberger based on the paper of Kenosi et al. (2015)^46^ may also support this idea. His hypothesis was “The degree of subsequent cerebral vasoconstriction might mainly be dependent on PaO_2_ levels”. However, such an interpretation should be made with caution, given the lack of sufficient metabolic parameters in such constrained experiments in neonates. Future recordings combining NIRS and diffuse correlation spectroscopy (DCS) could possibly provide information about changes in metabolism, as evaluated by the cerebral metabolic rate of oxygen (CMRO_2_)^31^. Similarly, a decrease in pH (increase in H^+^ and PaCO_2_) and increase in intracellular bicarbonate concentration decreases the threshold of neuronal excitability^47,48^. This is likely to increase oxygen extraction and the TOI in the “Lièvre” group more than in the “Tortue” group. The mechanisms responsible are complex and we have only mentioned the most obvious. The mode of delivery, for example, can also be considered. The three children born by elective Caesarean section were in the “Tortue” group. Thus, they did not undergo the same pre-clamping stress; their pH and BE were high and their lactate levels low. They were in a situation of a gradual, slower increase in the TOI. Regardless of the degree of stress, which is moderate in a situation of normal childbirth, both strategies allow reaching similar TOI values 10 min after birth. This highlights the strong attraction of the pivotal hemodynamic value of TOI at 10 min after birth and the plasticity of the adaptative mechanisms to reach this reference TOI value.

One limitation of this study was that we did not evaluate other metabolic mechanisms that could have influenced tissue oxygenation. Autoregulation involves other metabolic factors, such as (1) glucose levels^49^, for which both a negative and positive correlation with fractional tissue oxygen extraction have been described during the neonatal transition^50^ and (2) temperature, which may also modify the affinity of oxygen to red blood cells, even if the still immature thermoregulation of the neonate limits such a Bohr effect^51^.

Studying cerebral hemodynamic activity provides complementary information about the strategies used by the neuronal and vascular systems in their physiological adaptation to extrauterine life. We identified two main strategies, one consisting of a rapid increase in TOI within the first 6 min, whereas the second is slower. The two strategies correlated with the initial value of pH, BE, and lactate. Consistent with the good prognostic value of an SpO_2_ > 80% at 5 min of life^52^, it would be highly useful to evaluate to what extent the initial slope of the TOI could be predictive in pathological situations, such as perinatal anoxo-ischemia. This should be tested in a larger cohort that includes normal and pathological neonates with per partum anoxic stress. Additionally, the precise coupling between brain hemodynamics and neural activity has been the subject of numerous physiological studies in both adults^53^ and premature infants^30,31^, and in pathological situations in humans^54^ (and in animals^55,56^). Simultaneous multimodal analysis, combining EEG and NIRS, on the one hand, and systemic and ventilator parameters, on the other, would provide a better understanding of the interactions between cerebral oxygenation and neuronal activity during delivery.

## Methods

### Subjects

Twenty term neonates (11 females, mean gestational age (GA) at birth: 39.8 weeks GA (wGA) [38.6–41.1 wGA]) were tested in the supine position (recording onset time: 1.75 min of life, Tables 1 and 2). The study was approved by an ethics committee (Comités de protection des personnes (CPP) Ile-de-France VII, PI2019_843_0031) according to the guidelines of the Declaration of Helsinki of 1975. As this was an initial exploratory pilot study, a maximum of 30 patients was allowed to be included. Parents were informed about the study and provided their informed consent within 24 h prior to birth. The inclusion criteria were healthy full-term singleton neonates who showed a good neonatal transition to extrauterine life. Neonates with a suspicion of congenital malformation evaluated by antenatal echography were not included. Neonates were excluded if they required resuscitation in the first 10 min of life or displayed a congenital malformation.

### Data acquisition

Similar to our previous study^31^, a continuous wave (CW) near-infrared spectroscopy probe NIRO-200NX (Hamamatsu Photonics Corp., Tokyo, Japan) was placed on the infant’s forehead to evaluate cerebral tissue oxygenation. The NIRO 200 NX uses spatially resolved spectroscopy at three wavelengths (λ = 735, 810, and 850 nm). It is based on the solution of the diffusion approximation equation for a highly scattering semi-infinite homogeneous medium^31^. The effective light attenuation coefficient can be estimated by measuring the decrease in reflected light as a function of distance^31^. By assuming wavelength dependence of the reduced scattering coefficient, the spectral shape of the absorption coefficient can then be calculated and the cerebral TOI estimated^57^. The average output power of the lasers was less than 2 mW, which is under the nociceptive threshold^58^. The CW acquisition rate was set at 5 Hz (200 ms). The changes in TOI ([HbO]/[HbO] + [HbR]), oxyhemoglobin [∆HbO], and deoxyhemoglobin [∆HbR] were measured. The hemoglobin difference ([∆HbD] = [∆HbO] − [∆HbR]) and total hemoglobin ([∆HbT] = [∆HbO] + [∆HbR]) were calculated. The sensor was comprised of one emitter and two detectors. The probes were fixed in a holder to maintain a fixed distance of 3 cm between the emitters and detectors^31,59^. To attach the sensor as rapidly as possible and avoid mobilization of the neonate, a tubular bandage elastic net (Surgifix®) was used to cover the baby's head to which the sensor had been previously attached. This tubular net was covered by a second layer consisting of a jersey net, which is generally used to cover the neonate's head to avoid thermal loss (See illustration 1B).

### Recording procedures

We paid particular attention to avoid modifying the care of the neonate. At birth, the “skin-to-skin” procedure was applied after vaginal delivery. In cases of C-section, the baby was put on a resuscitation table under a radiant warmer. The probe was quickly placed on the hairless frontal area, over the forehead, parallel to the eyebrows (Fig. 1A). The recording started as soon as the neonate made its first cry, the very first clinical index of effective breathing and adaptation to extra uterine life^60^, generally within 2 min after delivery. Whole-body delivery was considered as T0. The monitoring stopped 10 min after birth. The probe, with the nets, was easily and quickly removed. The baby's standard care was not modified (Apgar scoring, cord gas, etc.) during the monitoring procedure. All clinical and paraclinical parameters were monitored by the midwife following standard procedures, in parallel to the recording.

### Data analysis

Off-line analysis of all signals was performed using in-house MATLAB scripts (Release 2018b, The MathWorks, Inc., Natick, Massachusetts, United States).

The time between delivery (Expulsion: T0) and the start of recording varied slightly between newborns. All recordings were time-aligned to T0.

An artefact-rejection procedure was developed. Extreme TOI values < 45% and > 90% were rejected. A moving window was used to extract the mean signal in periods of 2 s. Signals higher than two standard deviations were considered to be artefacts^61^ and rejected. If > 50% of the signal was rejected, the entire recording was rejected for the rest of analysis. The remaining hemodynamic signals were splined to substitute for the rejected data. The signals were then filtered using the Savitzky-Golay algorithm (order 3, window = 12 s after optimization) to further eliminate spurious, rapid, unrelated noise. After application of the Kolmogorof-Smirnov test for normality, the hemodynamic responses ([∆HbO], [∆HbR], [∆HbT], [∆HbD]), and TOI were then averaged across all subjects (time window: [0 to 10 min]), as tissular cerebral oxygenation was the same for all infants, regardless of the mode of delivery^33^.

To evaluate the global dynamics of all hemodynamic parameters, two analyses were applied to every hemodynamic parameter: (1) the first consisted of an ANOVA with a Friedman test to compare their evolution, minute by minute, supplemented by a post-hoc Durbin–Conover test, using jamovi software (jamovi version 1.6.23., https://www.jamovi.org) and (2) the second, comparison to a reference value using a t test.

1. The comparison was based on the mean of the parameter for each minute according to subject by non-parametric ANOVA for repeated measurements with a Friedman test. For threshold *p*-values < 0.05, a post-hoc Durbin–Conover test was applied to compare pairs (each minute was named M + the number of minutes.
2. The trends of the various parameters were monitored by performing statistical analyses (*student t-test*) on the means of the extracted values of the hemodynamic signals for each timepoint according to a reference value (for TOI, the mean of the previous 2 min [considered as a stable state during this period] and the other NIRS parameters, zero as a reference). A deflection point was then defined on the mean curve at the transition from significant to non-significant changes in TOI, the significance being evaluated from the immediate previous period (1 min).

### Evaluation of signal dynamics

The various curves were fitted to an exponential equation to better define the dynamics of the amplitude changes in TOI according to the time from delivery. Four points were then extracted (τ1, τ2, τ3, and τ4), corresponding to the time to reach 63.2, 86.5, 95, and 98.2% of the maximal value of the TOI, respectively (Fig. S1). The initial dynamics were monitored by calculating the slope between τ1 and τ2 based on the linear aspect of the curve during this period. The time to reach 95% of the maximum TOI (τ3) was plotted against the slope of the TOI between τ1 and τ2. A clustering approach was developed to search for homogeneous populations with similar trends based on the distribution of the resulting curves. Clustering using the Davies-Bouldin method indicates the optimal number of clusters. After identifying the number of clusters, clustering was automatically performed using the Matlab® *“k-means”* function*.* Finally, we assessed the clinical and paraclinical information for their predictive value for the extrauterine transition by comparing the clustered populations with their clinical and paraclinical neonatal parameters using the Mann–Whitney test (maternal age, duration of labor, duration of expulsive efforts [DEE], GA, birth weight, cord gas [pH, lactic acid, BE, pCO_2_]). If a clinical or paraclinical parameter was different according to group, the correlation between τ3 and the parameter value was studied by automatic clustering.

## Supplementary Information

Supplementary Information 1.

## References

[1] Marshall S, Lang AM, Perez M, Saugstad OD (2019). Delivery room handling of the newborn. J. Perinat. Med..

[2] Hagberg B, Hagberg G, Beckung E, Uvebrant P (2001). Changing panorama of cerebral palsy in Sweden. VIII. Prevalence and origin in the birth year period 1991–94. Acta Paediatr..

[3] Clark SL, Hankins GDV (2003). Temporal and demographic trends in cerebral palsy—Fact and fiction. Am. J. Obstet. Gynecol..

[4] Singer D (1999). Neonatal tolerance to hypoxia: A comparative-physiological approach. Comp. Biochem. Physiol. A Mol. Integr. Physiol..

[5] Vrancken SL, van Heijst AF, de Boode WP (2018). Neonatal hemodynamics: From developmental physiology to comprehensive monitoring. Front. Pediatr..

[6] Hooper SB, Roberts C, Dekker J, te Pas AB (2019). Issues in cardiopulmonary transition at birth. Semin. Fetal Neonatal Med..

[7] Riviere D, McKinlay CJD, Bloomfield FH (2017). Adaptation for life after birth: A review of neonatal physiology. Anaesth. Intensive Care Med..

[8] Connors G (1992). Perinatal assessment of cerebral flow velocity wave forms in the human fetus and neonate. Pediatr. Res..

[9] Madar J (2021). European resuscitation council guidelines 2021: Newborn resuscitation and support of transition of infants at birth. Resuscitation.

[10] Dawson JA (2010). Defining the reference range for oxygen saturation for infants after birth. Pediatrics.

[11] Iliodromiti S, Mackay DF, Smith GCS, Pell JP, Nelson SM (2014). Apgar score and the risk of cause-specific infant mortality: A population-based cohort study. Lancet.

[12] Papile LA (2001). The Apgar score in the 21st century. N. Engl. J. Med..

[13] James LS, Weisbrot IM, Prince CE, Holaday DA, Apgar V (1958). The acid–base status of human infants in relation to birth asphyxia and the onset of respiration. J. Pediatr..

[14] Malin GL, Morris RK, Khan KS (2010). Strength of association between umbilical cord pH and perinatal and long term outcomes: Systematic review and meta-analysis. BMJ.

[15] Manley BJ (2017). Towards evidence-based resuscitation of the newborn infant. Lancet.

[16] Noori S (2012). Transitional changes in cardiac and cerebral hemodynamics in term neonates at birth. J. Pediatr..

[17] Fauchère J-C (2010). Near-infrared spectroscopy measurements of cerebral oxygenation in newborns during immediate postnatal adaptation. J. Pediatr..

[18] Bruckner M, Pichler G, Urlesberger B (2020). NIRS in the fetal to neonatal transition and immediate postnatal period. Semin. Fetal Neonatal Med..

[19] Pichler G (2013). Reference ranges for regional cerebral tissue oxygen saturation and fractional oxygen extraction in neonates during immediate transition after birth. J. Pediatr..

[20] Urlesberger B (2010). Regional oxygen saturation of the brain and peripheral tissue during birth transition of term infants. J. Pediatr..

[21] Carli AD (2019). Cerebral oxygenation and blood flow in term infants during postnatal transition: BabyLux project. Arch. Dis. Child. Fetal Neonatal Ed..

[22] Morton S, Brodsky D (2017). Fetal physiology and the transition to extrauterine life. Clin. Perinatol..

[23] Schwaberger B (2018). Cerebral blood volume during neonatal transition in term and preterm infants with and without respiratory support. Front. Pediatr..

[24] Rainaldi MA, Perlman JM (2016). Pathophysiology of birth asphyxia. Clin. Perinatol..

[25] Pérez MLM, Hernández Garre JM, Pérez PE (2021). Analysis of factors associated with variability and acidosis of the umbilical artery pH at birth. Front. Pediatr..

[26] Baik N (2015). Reference ranges for cerebral tissue oxygen saturation index in term neonates during immediate neonatal transition after birth. Neonatology.

[27] Willie CK, Tzeng Y-C, Fisher JA, Ainslie PN (2014). Integrative regulation of human brain blood flow. J. Physiol..

[28] Yoon S, Zuccarello M, Rapoport RM (2012). pCO_2_ and pH regulation of cerebral blood flow. Front. Physiol..

[29] Schwaberger B, Pichler G, Urlesberger B (2016). Does cerebral vasoconstriction following delivery protect against hyperoxia?. J. Pediatr..

[30] Mahmoudzadeh M (2013). Syllabic discrimination in premature human infants prior to complete formation of cortical layers. Proc. Natl. Acad. Sci..

[31] Nourhashemi M, Mahmoudzadeh M, Goudjil S, Kongolo G, Wallois F (2020). Neurovascular coupling in the developing neonatal brain at rest. Hum. Brain Mapp..

[32] Schwaberger B (2014). Even mild respiratory distress alters tissue oxygenation significantly in preterm infants during neonatal transition. Physiol. Meas..

[33] Almaazmi M (2013). Cerebral near-infrared spectroscopy during transition of healthy term newborns. Neonatology.

[34] Katz M, Lunenfeld E, Meizner I, Bashan N, Gross J (1987). The effect of the duration of the second stage of labour on the acid–base state of the fetus. BJOG Int. J. Obstet. Gynaecol..

[35] Uzan S, Berkane N, Verstraete L, Mathieu E, Bréart G (2003). Acid base balance in the fetus during labor: Pathophysiology and exploration methods. J. Gynecol. Obstet. Biol. Reprod. (Paris).

[36] Aldrich CJ (1996). Fetal heart rate changes and cerebral oxygenation measured by nearinfrared spectroscopy during the first stage of labour. Eur. J. Obstet. Gynecol. Reprod. Biol..

[37] Berger R, Jensen A, Krieglstein J, Steigelmann JP (1991). Effects of acute asphyxia on brain energy metabolism in fetal guinea pigs near term. J. Dev. Physiol..

[38] Haworth SG, Hislop AA (1981). Adaptation of the pulmonary circulation to extra-uterine life in the pig and its relevance to the human infant. Cardiovasc. Res..

[39] Aldrich CJ (1994). Fetal cerebral oxygenation measured by near-infrared spectroscopy shortly before birth and acid–base status at birth. Obstet. Gynecol..

[40] Bhutani VK (1997). Extrauterine adaptations in the newborn. Semin. Neonatol..

[41] Morimoto A (2019). Measurement of the absolute value of cerebral blood volume and optical properties in term neonates immediately after birth using near-infrared time-resolved spectroscopy: A preliminary observation study. Appl. Sci..

[42] Schwaberger B (2015). Transitional changes in cerebral blood volume at birth. Neonatology.

[43] Liu Y, Harder DR, Lombard JH (2002). Interaction of myogenic mechanisms and hypoxic dilation in rat middle cerebral arteries. Am. J. Physiol.-Heart Circ. Physiol..

[44] Tamussino A (2016). Low cerebral activity and cerebral oxygenation during immediate transition in term neonates—A prospective observational study. Resuscitation.

[45] Amigoni A (2011). Four-side near-infrared spectroscopy measured in a paediatric population during surgery for congenital heart disease. Interact. Cardiovasc. Thorac. Surg..

[46] Kenosi M (2015). Effects of fractional inspired oxygen on cerebral oxygenation in preterm infants following delivery. J. Pediatr..

[47] Ruffin VA, Salameh AI, Boron WF, Parker MD (2014). Intracellular pH regulation by acid–base transporters in mammalian neurons. Front. Physiol..

[48] Jones RT, Faas GC, Mody I (2014). Intracellular bicarbonate regulates action potential generation via KCNQ channel modulation. J. Neurosci..

[49] Mattersberger C, Schmölzer GM, Urlesberger B, Pichler G (2020). Blood glucose and lactate levels and cerebral oxygenation in preterm and term neonates—A systematic qualitative review of the literature. Front. Pediatr..

[50] Matterberger C (2018). Blood glucose and cerebral tissue oxygenation immediately after birth—An observational study. J. Pediatr..

[51] Sasagawa K, Imai K, Kobayashi M (2006). Influence of allosteric effectors and temperature on oxygen binding properties and the Bohr effect of bovine hemoglobin. Zool. Sci..

[52] Andresen B (2019). Cerebral oxygenation and blood flow in normal term infants at rest measured by a hybrid near-infrared device (BabyLux). Pediatr. Res..

[53] Raichle ME (1998). Behind the scenes of functional brain imaging: A historical and physiological perspective. Proc. Natl. Acad. Sci. U. S. A..

[54] Bourel-Ponchel E, Mahmoudzadeh M, Delignières A, Berquin P, Wallois F (2017). Non-invasive, multimodal analysis of cortical activity, blood volume and neurovascular coupling in infantile spasms using EEG-fNIRS monitoring. NeuroImage Clin..

[55] Osharina V, Ponchel E, Aarabi A, Grebe R, Wallois F (2010). Local haemodynamic changes preceding interictal spikes: A simultaneous electrocorticography (ECoG) and near-infrared spectroscopy (NIRS) analysis in rats. Neuroimage.

[56] Mahmoudzadeh M, Dehaene-Lambertz G, Wallois F (2017). Electrophysiological and hemodynamic mismatch responses in rats listening to human speech syllables. PLoS ONE.

[57] Matcher SJ, Elwell CE, Cooper CE, Cope M, Delpy DT (1995). Performance comparison of several published tissue near-infrared spectroscopy algorithms. Anal. Biochem..

[58] Nourhashemi M, Mahmoudzadeh M, Wallois F (2016). Thermal impact of near-infrared laser in advanced noninvasive optical brain imaging. Neurophotonics.

[59] Quaresima V, Ferrari M (2019). Functional near-infrared spectroscopy (fNIRS) for assessing cerebral cortex function during human behavior in natural/social situations: A concise review. Organ. Res. Methods.

[60] Ashish KC (2020). Not crying after birth as a predictor of not breathing. Pediatrics.

[61] Di Lorenzo R (2019). Recommendations for motion correction of infant fNIRS data applicable to multiple data sets and acquisition systems. Neuroimage.

