# Supplementary material for: Dynamics of cortical oxygenation during immediate adaptation to extrauterine life
Source: Sci Rep. 2021 Nov 11;11:22041. doi: 10.1038/s41598-021-01674-9 (PMC8586152; doi:10.1038/s41598-021-01674-9)
Supplement: Supplementary file 1 — Supplementary Information 1. [file 41598_2021_1674_MOESM1_ESM.docx]

**Supplementary Materials**

**Time constant**


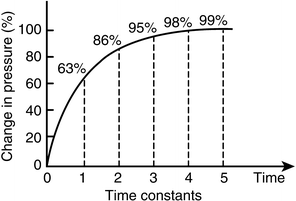
The time constant τ (tau), was adapted from the physics and engineering domains^1^ and is now used in several scientific domains (neuroscience^2^, physiology^3^, meteorology^4^, etc.). The time constant is the time for the response of the system to reach 63.2% of its final (asymptotic) value.

The value of 63.2% comes from the equation:

- *Percentage* *of* *change* = (1 − 1/*e^t^*^/^*^τ^*) ×100%

When t/τ = 1 (one time constant), the value is equal to:

- *Percentage* *of* *change* = (1 − 1/*e*^1^) × 100% = 63.2%

For two-time constants, the value is equal to:

- *Percentage* *of* *change* = (1−1/*e*^2^) × 100% = 86.47%

After four-time constants, the response of the system reaches 98% of its final value.


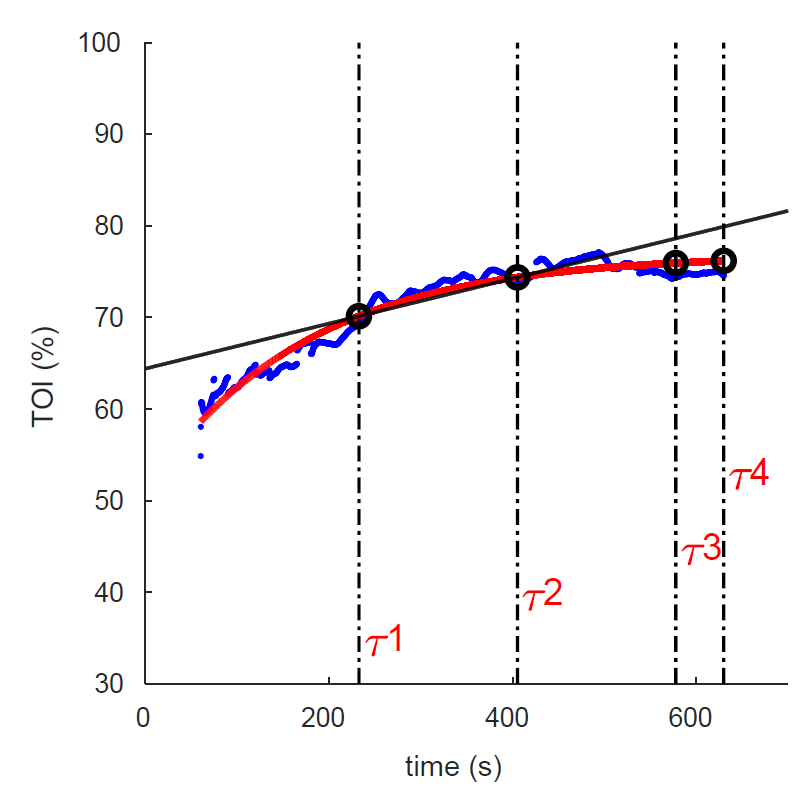


Figure S1. Sample TOI data from one subject (in blue), with the fitted curve (in red) and the linear regression line (in black). The positions of markers τ1, τ2, τ3, and τ4, corresponding to 63.2%, 86.5%, 95%, and 98.2% of the maximal value of the TOI, respectively, are shown on the curves.

**Artefact rejection**

After artefact rejection, the median percentage of extreme values was 2.33 [0.66 – 7.27]. The median percentage of values > 2 SD was 1.91 [0.44-4.02] for TOI, 1.60 [1.29 – 2.32] for [ΔHbO], and 1.60 [0.75 – 2.8] for [ΔHbR]. Two entire recordings were rejected.

Table S1. Recorded data points and data points rejected for each measurement

|  |  | Extreme values (TOI <45 & TOI >90) | | Values > 2SD with window meaning | | | | | |
| --- | --- | --- | --- | --- | --- | --- | --- | --- | --- |
|  |  | **TOI** | | **TOI** | | **ΔHbO** | | **ΔHbR** | |
|  | Recording samples | **Number of samples** | **Percentage of samples** | **Number of samples** | **Percentage of samples** | **Number of samples** | **Percentage of samples** | **Number of samples** | **Percentage of samples** |
| sub001 | 2250 | 21 | 0.93 | 90 | 4.00 | 35 | 1.56 | 131 | 5.82 |
| sub002 | 2850 | 20 | 0.70 | 1 | 0.04 | 3 | 0.11 | 5 | 0.18 |
| sub003 | 2250 | 222 | 9.87 | 286 | 12.71 | 69 | 3.07 | 39 | 1.73 |
| sub004 | 2780 | 6 | 0.22 | 30 | 1.08 | 19 | 0.68 | 38 | 1.37 |
| sub005 | 2325 | 1294 | 55.66 | 5 | 0.22 | 47 | 2.02 | 207 | 8.90 |
| sub006 | 2775 | 3 | 0.11 | 193 | 6.95 | 40 | 1.44 | 138 | 4.97 |
| sub007 | 2775 | 73 | 2.63 | 158 | 5.69 | 62 | 2.23 | 51 | 1.84 |
| sub008 | 1650 | 20 | 1.21 | 126 | 7.64 | 59 | 3.58 | 45 | 2.73 |
| sub009 | 2775 | 122 | 4.40 | 61 | 2.20 | 37 | 1.33 | 8 | 0.29 |
| sub010 | 2850 | 15 | 0.53 | 108 | 3.79 | 8 | 0.28 | 22 | 0.77 |
| sub011 | 2850 | 58 | 2.04 | 116 | 4.07 | 42 | 1.47 | 101 | 3.54 |
| sub012 | 2314 | 508 | 21.95 | 0 | 0.00 | 27 | 1.17 | 37 | 1.60 |
| sub013 | 1973 | 0 | 0.00 | 0 | 0.00 | 51 | 2.58 | 17 | 0.86 |
| sub014 | 1528 | 1216 | 79.58 | 8 | 0.52 | 49 | 3.21 | 46 | 3.01 |
| sub015 | 2475 | 11 | 0.44 | 16 | 0.65 | 8 | 0.32 | 39 | 1.58 |
| sub016 | 2550 | 97 | 3.80 | 0 | 0.00 | 42 | 1.65 | 18 | 0.71 |
| sub017 | 2168 | 139 | 6.41 | 35 | 1.61 | 31 | 1.43 | 37 | 1.71 |
| sub018 | 2700 | 47 | 1.74 | 86 | 3.19 | 47 | 1.74 | 43 | 1.59 |
| sub019 | 2550 | 67 | 2.63 | 59 | 2.31 | 44 | 1.73 | 18 | 0.71 |
| sub020 | 2700 | 284 | 10.52 | 44 | 1.63 | 140 | 5.19 | 2 | 0.07 |
| Median | **2550.00** | **62.50** | **2.33** | **51.50** | **1.91** | **42.00** | **1.60** | **38.50** | **1.60** |
| First Quartile (Q1) | **2250** | **18.75** | **0.65** | **7.25** | **0.44** | **30** | **1.29** | **18** | **0.75** |
| Third Quartile (Q3) | **2775** | **159.75** | **7.27** | **110** | **4.01** | **49.5** | **2.32** | **47.25** | **2.79** |

**Statistical analyses**

|  | Hemodynamic parameters | Statistical results (Post hoc test) |
| --- | --- | --- |
| TOI  (%) |  | 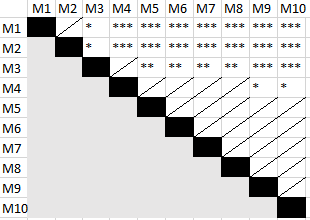 |
| ∆HbO  (a.u.) |  | *Non applicable* |
| ∆HbR  (a.u.) |  | 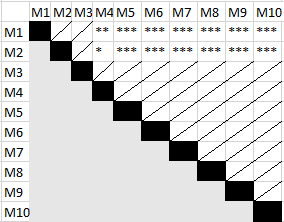 |
| ∆HbD  (a.u.) |  | 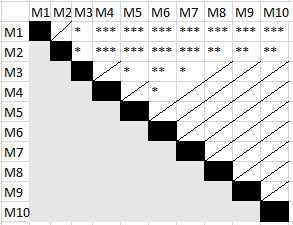 |
| ∆HbT  (a.u.) |  | 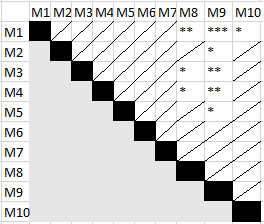 |

Figure S2. In the first column, the hemodynamic parameters TOI, [ΔHbO], [ΔHbR], [ΔHbD], and [ΔHbT] are presented as boxplots of window periods of 1 bin (e.g.: M1 corresponds to the first bin (first minute of recording). The second column presents the statistical results of the post-hoc Durbin-Conover test after the Friedman test. *p < 0.05, ** p < 0.01, ***p < 0.001

Table S2. This table summarizes the statistical results of the ANOVA with the Friedman test for each hemodynamic parameter (TOI, [ΔHbO], [ΔHbR], [ΔHbD], and [ΔHbT]) with the p-value (*p < 0.05, ** p < 0.01, ***p < 0.001).

|  | Friedman results (df = 9) | | |
| --- | --- | --- | --- |
| Parameters | **χ²** | **p-value** | |
| TOI | 40.5 | < 0 .001 | *** |
| ∆HbO | 7.36 | 0.599 |  |
| ∆HbR | 48.8 | < 0 .001 | *** |
| ∆HbD | 33.5 | < 0 .001 | *** |
| ∆HbT | 17.7 | 0.038 | * |

Table S3. This table summarizes the detailed results for all post hoc test for each pair of periods for the hemodynamic parameters TOI, [ΔHbR], [ΔHbD], and [ΔHbT]. *p < 0.05, **p < 0.01, ***p < 0.001 ; Stat. : Statistic

| Pair | | **TOI** | | | **[∆HbR]** | | | **[∆HbD]** | | | **[∆HbT]** | | |
| --- | --- | --- | --- | --- | --- | --- | --- | --- | --- | --- | --- | --- | --- |
| Comparing bins | | ***Stat.*** | ***p-value*** | | ***Stat.*** | ***p-value*** | | ***Stat.*** | ***p-value*** | | ***Stat.*** | ***p-value*** | |
| ***M1*** | ***M2*** | 0.137 | 0.892 |  | 1.204 | 0.234 |  | 0.598 | 0.553 |  | 0.964 | 0.339 |  |
| ***M1*** | ***M3*** | 2.189 | 0.033 | * | 1.549 | 0.127 |  | 2.630 | 0.011 | * | 0.675 | 0.503 |  |
| ***M1*** | ***M4*** | 3.831 | < .001 | *** | 3.269 | 0.002 | ** | 3.705 | < .001 | *** | 0.578 | 0.565 |  |
| ***M1*** | ***M5*** | 4.926 | < .001 | *** | 6.194 | < .001 | *** | 4.781 | < .001 | *** | 1.446 | 0.154 |  |
| ***M1*** | ***M6*** | 4.926 | < .001 | *** | 7.399 | < .001 | *** | 5.737 | < .001 | *** | 1.928 | 0.059 |  |
| ***M1*** | ***M7*** | 5.610 | < .001 | *** | 8.087 | < .001 | *** | 5.020 | < .001 | *** | 1.928 | 0.059 |  |
| ***M1*** | ***M8*** | 5.200 | < .001 | *** | 7.915 | < .001 | *** | 3.825 | < .001 | *** | 2.796 | 0.007 | ** |
| ***M1*** | ***M9*** | 5.884 | < .001 | *** | 7.571 | < .001 | *** | 3.944 | < .001 | *** | 3.567 | < .001 | *** |
| ***M1*** | ***M10*** | 6.294 | < .001 | *** | 7.571 | < .001 | *** | 3.825 | < .001 | *** | 2.024 | 0.048 | * |
| ***M2*** | ***M3*** | 2.053 | 0.045 | * | 0.344 | 0.732 |  | 2.032 | 0.047 | * | 0.289 | 0.773 |  |
| ***M2*** | ***M4*** | 3.695 | < .001 | *** | 2.065 | 0.044 | * | 3.108 | 0.003 | ** | 0.385 | 0.701 |  |
| ***M2*** | ***M5*** | 4.789 | < .001 | *** | 4.990 | < .001 | *** | 4.183 | < .001 | *** | 0.482 | 0.632 |  |
| ***M2*** | ***M6*** | 4.789 | < .001 | *** | 6.194 | < .001 | *** | 5.139 | < .001 | *** | 0.964 | 0.339 |  |
| ***M2*** | ***M7*** | 5.473 | < .001 | *** | 6.882 | < .001 | *** | 4.422 | < .001 | *** | 0.964 | 0.339 |  |
| ***M2*** | ***M8*** | 5.063 | < .001 | *** | 6.710 | < .001 | *** | 3.227 | 0.002 | ** | 1.832 | 0.072 |  |
| ***M2*** | ***M9*** | 5.747 | < .001 | *** | 6.366 | < .001 | *** | 3.347 | 0.001 | ** | 2.603 | 0.012 | * |
| ***M2*** | ***M10*** | 6.158 | < .001 | *** | 6.366 | < .001 | *** | 3.227 | 0.002 | ** | 1.060 | 0.294 |  |
| ***M3*** | ***M4*** | 1.642 | 0.106 |  | 1.721 | 0.091 |  | 1.076 | 0.287 |  | 0.096 | 0.924 |  |
| ***M3*** | ***M5*** | 2.737 | 0.008 | ** | 4.646 | < .001 | *** | 2.151 | 0.036 | * | 0.771 | 0.444 |  |
| ***M3*** | ***M6*** | 2.737 | 0.008 | ** | 5.850 | < .001 | *** | 3.108 | 0.003 | ** | 1.253 | 0.215 |  |
| ***M3*** | ***M7*** | 3.421 | 0.001 | ** | 6.538 | < .001 | *** | 2.390 | 0.020 | * | 1.253 | 0.215 |  |
| ***M3*** | ***M8*** | 3.010 | 0.004 | ** | 6.366 | < .001 | *** | 1.195 | 0.237 |  | 2.121 | 0.039 | * |
| ***M3*** | ***M9*** | 3.695 | < .001 | *** | 6.022 | < .001 | *** | 1.315 | 0.194 |  | 2.892 | 0.005 | ** |
| ***M3*** | ***M10*** | 4.105 | < .001 | *** | 6.022 | < .001 | *** | 1.195 | 0.237 |  | 1.349 | 0.183 |  |
| ***M4*** | ***M5*** | 1.095 | 0.279 |  | 2.925 | 0.005 | ** | 1.076 | 0.287 |  | 0.867 | 0.389 |  |
| ***M4*** | ***M6*** | 1.095 | 0.279 |  | 4.129 | < .001 | *** | 2.032 | 0.047 | * | 1.349 | 0.183 |  |
| ***M4*** | ***M7*** | 1.779 | 0.081 |  | 4.818 | < .001 | *** | 1.315 | 0.194 |  | 1.349 | 0.183 |  |
| ***M4*** | ***M8*** | 1.368 | 0.177 |  | 4.646 | < .001 | *** | 0.120 | 0.905 |  | 2.217 | 0.031 | * |
| ***M4*** | ***M9*** | 2.053 | 0.045 | * | 4.302 | < .001 | *** | 0.239 | 0.812 |  | 2.989 | 0.004 | ** |
| ***M4*** | ***M10*** | 2.463 | 0.017 | * | 4.302 | < .001 | *** | 0.120 | 0.905 |  | 1.446 | 0.154 |  |
| ***M5*** | ***M6*** | 0.000 | 1.000 |  | 1.204 | 0.234 |  | 0.956 | 0.343 |  | 0.482 | 0.632 |  |
| ***M5*** | ***M7*** | 0.684 | 0.497 |  | 1.893 | 0.064 |  | 0.239 | 0.812 |  | 0.482 | 0.632 |  |
| ***M5*** | ***M8*** | 0.274 | 0.785 |  | 1.721 | 0.091 |  | 0.956 | 0.343 |  | 1.349 | 0.183 |  |
| ***M5*** | ***M9*** | 0.958 | 0.342 |  | 1.376 | 0.174 |  | 0.837 | 0.406 |  | 2.121 | 0.039 | * |
| ***M5*** | ***M10*** | 1.368 | 0.177 |  | 1.376 | 0.174 |  | 0.956 | 0.343 |  | 0.578 | 0.565 |  |
| ***M6*** | ***M7*** | 0.684 | 0.497 |  | 0.688 | 0.494 |  | 0.717 | 0.476 |  | 0.000 | 1.000 |  |
| ***M6*** | ***M8*** | 0.274 | 0.785 |  | 0.516 | 0.608 |  | 1.912 | 0.061 |  | 0.867 | 0.389 |  |
| ***M6*** | ***M9*** | 0.958 | 0.342 |  | 0.172 | 0.864 |  | 1.793 | 0.079 |  | 1.639 | 0.107 |  |
| ***M6*** | ***M10*** | 1.368 | 0.177 |  | 0.172 | 0.864 |  | 1.912 | 0.061 |  | 0.096 | 0.924 |  |
| ***M7*** | ***M8*** | 0.411 | 0.683 |  | 0.172 | 0.864 |  | 1.195 | 0.237 |  | 0.867 | 0.389 |  |
| ***M7*** | ***M9*** | 0.274 | 0.785 |  | 0.516 | 0.608 |  | 1.076 | 0.287 |  | 1.639 | 0.107 |  |
| ***M7*** | ***M10*** | 0.684 | 0.497 |  | 0.516 | 0.608 |  | 1.195 | 0.237 |  | 0.096 | 0.924 |  |
| ***M8*** | ***M9*** | 0.684 | 0.497 |  | 0.344 | 0.732 |  | 0.120 | 0.905 |  | 0.771 | 0.444 |  |
| ***M8*** | ***M10*** | 1.095 | 0.279 |  | 0.344 | 0.732 |  | 0.000 | 1.000 |  | 0.771 | 0.444 |  |
| ***M9*** | ***M10*** | 0.411 | 0.683 |  | 0.000 | 1.000 |  | 0.120 | 0.905 |  | 1.542 | 0.129 |  |

***References***

1. Daoud, E. G., Farag, H. L. & Chatburn, R. L. Airway Pressure Release Ventilation: What Do We Know? *Respir. Care* **57**, 282–292 (2012).

2. Isokawa, M. Membrane time constant as a tool to assess cell degeneration. *Brain Res. Protoc.* **1**, 114–116 (1997).

3. Carlo, W. A. & Ambalavanan, N. Conventional Mechanical Ventilation: Traditional and New Strategies. *Pediatr. Rev.* **20**, e117–e126 (1999).

4. Wagner, N. K. The Effect of the Time Constant of Radiosonde Sensors on the Measurement of Temperature and Humidity Discontinuities in the Atmosphere. *Bull. Am. Meteorol. Soc.* **42**, 317–321 (1961).
